# Clinical and epidemiological characterisation of neurofibromatosis type 1: Combined analysis of a reference hospital in Brazil and DataSUS

**DOI:** 10.1590/1678-4685-GMB-2024-0144

**Published:** 2025-04-25

**Authors:** Marina Gabriela da Silva Lins, Peterson de Jesus Morais, Júlia de Oliveira Martinho, Mariah Cristina Antunes do Nascimento, Ana Paula Simedan Vila, Márcia Maria Urbanin Castanhole-Nunes, Érika Cristina Pavarino, Eny Maria Goloni-Bertollo

**Affiliations:** 1 Faculdade de Medicina de São José do Rio Preto (FAMERP), Departamento de Biologia Molecular, Unidade de Pesquisa em Genética e Biologia Molecular (UPGEM), São José do Rio Preto, SP, Brazil.; 2 Universidade Estadual Paulista (UNESP), Instituto de Biociências, Letras e Ciências Exatas (IBILCE), Departamento de Biologia, São José do Rio Preto, SP, Brazil.

**Keywords:** Neurofibromatosis, neurofibroma, neoplasms, nervous system diseases, genetic diseases

## Abstract

Neurofibromatosis type 1 (NF1) is a syndrome triggered by mutations in the *NF1* gene, which alter the neurofibromin protein, a negative regulator of the *RAS* oncogenic pathway. Due to underreporting, the scarcity of studies on NF1 in Brazil and its importance in public health. This study aimed to assess the clinical and epidemiological characterisation of NF1 in a Reference Hospital in the country and DataSUS. The study analysed the electronic medical records of patients with NF1 and the DataSUS databases. The medical records showed a greater number of female, white and adult patients. There was a high frequency of clinical features adopted by the NIH consensus for the clinical diagnosis of the disease, such as CALMs, dermal neurofibromas and axillary/inguinal ephelides, bone and ophthalmological changes, in addition malignant and benign neoplasms and neurodevelopmental disorders. On the other hand, the data provided by DataSUS shows a disproportionate concentration of NF1 consultations between the country’s regions, with a low level of diagnoses of newborn with NF1 and a NF1 mortality rate of 3.06% in the population. There is therefore a need for new public policies on access to diagnosis, treatment and information about the disease for the Brazilian population.

## Introduction

Neurofibromatoses are a class of autosomal dominant diseases, divided into Neurofibromatosis type 1 (NF1; OMIM 162200) (OMIM, 1986), Neurofibromatosis type 2 (NF2) and Schwannomatosis. Of these, NF1 is the most common and has the highest risk of developing into malignant cancer (Pinna *et al*., 2015; Ly and Blakeley, 2019). It is considered to be a syndrome caused by mutations in the *NF1* gene, which encodes the neurofibromin protein, a negative regulator of the *RAS* oncogenic pathway. Loss of neurofibromin function and activation of the RAS results in increased proliferative activity (Eoli *et al*., 2019; Landry *et al*., 2021). *NF1* is a tumor suppressor gene, and loss of the wild-type allele is observed in malignancies associated with neurofibromatosis (Carrion *et al*., 2023). Because of this, individuals with NF1 are more likely to develop malignant or benign tumours in the nervous system, skin or bones (neurofibromas, optic nerve glioma, meningioma and malignant peripheral nerve sheath neoplasia) (Gutmann *et al*., 2017; Staedtke *et al*., 2017; Miraglia *et al*., 2020). 

NF1 is an autosomal dominant disease with penetrance is close to 100%, with an incidence of approximately 1 in every 2.500/3.000 individuals, and is one of the most frequent autosomal diseases in the world population (Uusitalo *et al*., 2015; Friedman, 2022). Half of the cases of the disease are caused by genetic inheritance factors, but they can also be caused by new mutations (Bayat and Bayat, 2020). The clinical characteristics of NF1 vary greatly between individuals. However, according to the *National Institute of Health (NIH) Consensus Conference*, individuals with NF1 must have two of the seven diagnostic criteria, such as six or more café-au-lait macules (CALMs), axillary or inguinal ephelides, two or more cutaneous or plexiform neurofibromas of different types, two or more lish nodules (LNS), a specific skeletal lesion, an optic glioma or a first-degree relative affected (NIH, 1988). Café-au-lait macules (CALMs) and ephelides are signs present in both NF1 and Legius Syndrome, so a differential diagnosis must be made (Legius *et al*., 2021).

The NF1 mutation may also be related to some neurodevelopmental disorders such as cognitive impairment of the speech and motor system, Attention Deficit Hyperactivity Disorder (ADHD), Autism Spectrum Disorder (ASD), dyslexia, as well as other clinical features such as epilepsy and chronic headache (migraine) (North *et al*., 1997; Hyman *et al*., 2005; Pecoraro *et al*., 2017; Vogel *et al*., 2017).

Given that NF1 is a syndrome with scarce data in Brazil and of public health importance, the aim of this study was to investigate epidemiological data and the clinical characterisation of patients with NF1 treated at a quaternary reference university hospital in the country. In addition to analysing Brazilian public health databases.

## Subjects and Methods

The retrospective cross-sectional study carried out analysed clinical and epidemiological data on patients with NF1 treated between 2010 and 2020 at a Brazilian quaternary referral hospital (Hospital de Base, n.d.), which treats individuals from more than 102 municipalities and most of the administrative regions of Brazil and Latin America. Data available from the country’s Unified Health System (DataSUS) from 2010 to February 2024, which issues general data on diseases and health procedures carried out in the country, was also analysed (Brazil, 2020). Therefore, the analysis of hospital and DataSUS data was evaluated in order to obtain the greatest amount of information on the syndrome analysed.

The hospital’s electronic records were searched using the International Statistical Classification of Diseases and Related Health Problems (ICD) “Q85.0” and with “Neurofibromatosis” and the “Neurofibroma” keywords, to find patients diagnosed with NF1. The results of the data from the Reference Hospital showed that 316 patients were obtained, of whom only 187 had their diagnosis confirmed by meeting the clinical criteria and five by molecular tests. Those whose diagnosis was not confirmed because they had insufficient clinical information in their electronic medical records were not included in the analysis of the results. NF1 diagnosis criteria was realized with the NIH (1988) and Legius *et al*. (2021) revised criteria. 

The number of cases, family history and clinical data were investigated. Among the information available, age, gender, ethnicity, location, clinical characteristics of the patients, number of deaths and mortality rate were evaluated. The clinical characteristics described were: dermal and plexiform neurofibromas, CALMs, axillary and/or inguinal ephelides, ocular and visual changes (i.e. low visual acuity, strabismus and amblyopia), bone changes (i.e. wing dysplasia of the sphenoid bone), pseudarthrosis, scoliosis, limb dysmetria, LNs, optic nerve glioma, macrocephaly, hydrocephalus, epilepsy, neurological disorders (chronic headache, attention deficit hyperactivity disorder, dyslexia), development of benign and malignant tumours (malignant neoplasm of the peripheral nerve sheath, meningioma, arachnoid cyst, haemangioma, pheochromocytoma and leiomyoma) and precocious puberty.

The data collected on the Department of Informatics of the Unified Health System (DataSUS) platform, was done by following the tabs: “Health Information (TABNET)”, “Health Care”, “Hospital Production (SIH/SUS)” and “SUS Hospital Procedures - Place of hospitalisation” by region. For the number of treatments, number of deaths and mortality rate, the Contents tab was used, clicking on “AIH approved”, “Deaths” and “Mortality rate”, following the procedures tab by “0303110082 - neurofibromatosis treatment”. Data on births with NF1 was collected using the following tabs: “Vital Statistics”, “Live Births - 1994”, “Anomaly or Congenital Defect in Live Births - SINASC”, “Diagnosis Number”, “ICD - Q850” (Brazil, 2012; Brazil, 2022). DataSUS is the country’s main public health database, but it has a high level of data scarcity. It is therefore an important tool for the study’s data analyses (Aguiar *et al*., 2022).

Statistical analyses were carried out using the Statistical Package for Social Sciences (SPSS) IBM 23rd version and GraphPad Prism software version 9. The statistical tests used were Pearson’s chi-square test and Fisher’s Exact Test. In all analyses, a P-value of less than 0.05 was considered statistically significant.

The study was approved by the Research Ethics Committee of the Faculty of Medicine of São José do Rio Preto under number 3.380.276.

## Results

When analyzing the Reference Hospital medical records of the 316 patients examined with suspected NF1, only 192 had a confirmed diagnosis of NF1, giving a prevalence of 60% per case assessed. The medical records revealed a greater number of female (57.8%), white (89.6%), adult (18y-39y) patients (40.6%) and only (9%) had completed higher education, see Table 1.

**Table 1:** Incidence and characterisation of the population treated at the hospital with NF1.

Population characteristics	n	%	P-value
**Biological sex**			<0.0030 **
Female	111	57.8	
Male	81	42.2	
**Ethnicity and colour**			<0.0001 ****
White	171	89.6	
Brown	12	6.0	
Black	5	2.6	
Data unavailable	4	2.0	
**Age**			<0.0001 ****
Children and adolescents (4-17y)	55	28.6	
Young people and adults (18-39y)	78	40.6	
Adults and seniors (40-83y)	58	30.2	
Data unavailable	1	0.5	
**Level of education**			<0.0001 ****
Illiterate	29	15.1	
Primary to secondary education	97	50.5	
University education	18	9.0	
Data unavailable	48	25.0	

*Significant p-value <0,05

The NF1 patients in the study presented the clinical features that diagnose the disease according to the NIH (1988) consensus. Dermal neurofibroma, the main feature of NF1, was present in 135 patients. Precocious puberty, a complication associated with the presence of optic nerve glioma, was present in three patients (1.6%). The clinical features and their frequency in the patients can be seen in Table 2.

**Table 2:** Frequency in patients with clinical characteristics adopted in the National Institute of Health Consensus (1988).

Clinical characteristics	n	%
CALM*	178	92.7
Dermal neurofibroma	135	70.3
Axillary and/or inguinal ephelides	127	66.1
Specific bone lesions	120	62.5
Family history	85	44.3
Lisch nodule	56	29.2
Plexiform neurofibroma	44	22.9
Glioma of the optic nerve	8	4.1

*CALM: café-au-lait macules

Of the 192 patients, 7.2% developed malignant neoplasms and 18.7% developed benign neoplasms. Various skeletal and ocular changes were also observed in the individuals, see Table 3.

**Table 3:** Malignant and benign neoplasms, bone and eye alterations present in patients with NF1.

Neoplasms and alterations	n	%
***Malignant neoplasms***	**14**	**7.2**
MPNST*	3	1.5
Dermal basal cell carcinoma	3	1.5
Unspecified carcinomas	2	1
Gastrointestinal stromal tumour	1	0.5
Ductal carcinoma	1	0.5
Papillary urothelial carcinoma	1	0.5
Dermal carcinoma unspecified	1	0.5
Dermal squamous cell carcinoma	1	0.5
Papillary thyroid carcinoma	1	0.5
***Benign neoplasms***	**36**	**18.75**
Central nervous system cancer	29	15.1
Pheochromocytoma	2	1
Hemangioma	2	1
Meningioma	1	0.5
Arachnoid cyst	1	0.5
Thyroglossal duct cysts	1	0.5
***Ophthalmic alterations***	**28**	**14.5**
Low visual acuity	22	11.45
Strabismus	5	2.6
Amblyopia	1	0.5
***Skeletal alterations***	**120**	**62.5**
Scoliosis	59	30.7
Pectus excavatum	16	8.3
Hyperkyphosis	15	7.8
Hyperlordosis	6	3.1
Lower limb dysmetria	5	2.6
Flat feet	4	2.1
Genu Valgum	4	2.1
Congenital clubfoot	3	1.5
Pseudoarthrosis	3	1.5
Sphenoid wing dysplasia	3	1.5
Pectus Carinatum	1	0.5
Genu varus	1	0.5

*MPNST: Malignant peripheral nerve sheath tumour

Nuclear magnetic resonance imaging (MRI) was available in 55 patients and showed hyperintense T2-weighted images in 29 scans, giving an average of 52.7% of the patients examined with a positive result. The most common anatomical locations of these central nervous system lesions were the basal ganglia, cerebellum and trunk. 

Neurodevelopmental disorders were present in 35 patients, with an average of 18.2% among NF1 patients. The proportion of women and men with neurodevelopmental disorders was not very different overall, with 17 women and 18 men. However, there were differences in the proportions between the sexes, see Table 4.

**Table 4:** Frequency of neurodevelopmental disorders in the sexes of patients with NF1.

Neurodevelopmental disorders	Male			Female			P-value
	n	P	%	n	P	%	
Cognitive impairment	7	81	8.6	12	111	10.8	0.8074
Speech impairment	7		8.6	6		5.4	0.3981
Motor impairment	5		6.1	3		2.7	0.2854
ADHD	7		8.6	4		3.6	0.2077
ASD	4		4.9	0		0	0.0303*
Dyslexia	2		2.4	0		0	0.1767

Note: P, population; ADHD, attention deficit hyperactivity disorder; ASD, autism spectrum disorder. *Significant p-value <0,05 (Some patients had more than one associated disorder)

Epilepsy was diagnosed in five patients, an average of 2.6%, three of whom had focal epilepsy. Chronic headaches were reported by 24 patients (12.5%), with migraines predominating in 16 (8.4%) and tension headaches in six (3.1%). Macrocephaly was present in five (2.6%) and hydrocephalus in two (1%) patients.

**Figure 1:**
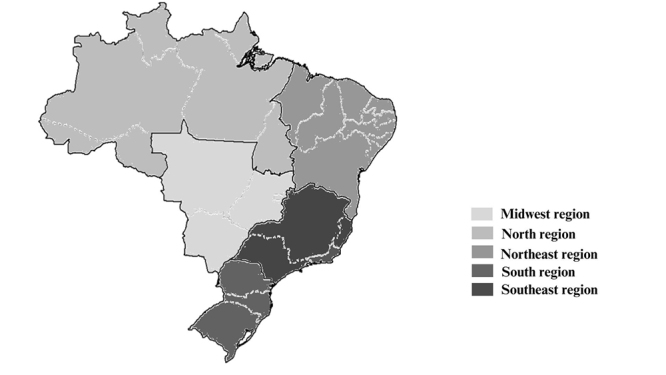
Number of treatments for neurofibromatosis in Brazilian regions from 2010 to 2024, according to DataSUS. The registration of NF1 cases number varies between light grey (n=73) to black colour (n=465).

The DataSUS database showed that 1145 people were treated for neurofibromatosis in health centres. The region with the highest number of cases was the Southeast with 465, while the other Brazilian regions were the South with 279, the Northeast with 250, the North with 78 and the Centre-West with 73 (Figure 1). The number of diagnoses of live births with neurofibromatosis was 22 individuals between 2010 and 2022, with an average of two individuals per year. 

The number of deaths from neurofibromatosis in Brazil from 2010 to 2024 was 35, with the South and Southeast regions having the highest number of deaths, with 11 and 13, respectively. The country’s mortality rate for NF1 is 3.06% of the population. 

## Discussion

The difficulty in diagnosing patients and the lack of knowledge among health professionals about neurofibromatosis type I (NF1) resulted in 40% of undiagnosed cases. It is therefore necessary to broaden the understanding of these professionals, and molecular tests, which are not available in Brazilian public health, could help with the genotype-phenotype relationship and patient follow-up. This deficit reduces the depth and attention of the clinical investigation of the disease (Iriart *et al*., 2019). 

Studies show that NF1 has the same incidence between genders and ethnicities (Friedman, 1999; Ly and Blakeley, 2019). However, the present study observed a higher incidence of NF1 in females and in white people. In agreement with our study, Landry *et al* (2021) reported that 52.3% of patients were female. These results are related to the specificity of the population studied, since men seek health services less than women and the country’s dark-skinned population is the most affected by poverty, lack of information and access to health services (Levorato *et al*., 2014; Silva *et al*., 2020). These factors are intrinsic to the results on gender, ethnicity and the incidence of NF1 in the population studied. 

The Brazilian regions with the highest frequency of care for NF1 were the South and Southeast, related to the concentration of public health care in these regions (Oliveira *et al*., 2019). On the other hand, the North and Centre-West regions had a lower number of patients treated, as they are regions with geographical barriers, limited access to healthcare and a lack of public policies (Oliveira *et al*., 2019).

The results showed that 35 people in the country died from NF1, with a mortality rate of 3.06%. According to the literature, patients with NF1 have their life expectancy reduced by up to 15 years compared to the general population (Rasmussen *et al*., 2001). Malignant tumours and vascular diseases are the main factors associated with death in these patients, with a higher frequency in patients under the age of 40 (Landry *et al*., 2021). 

The number of children born with NF1 in Brazil between 2010 and 2022 was 22 individuals. This low number is justified because children with NF1 show few characteristics of the disease during the early stages of development and only around 50% have familial history. NF1 is a progressive disease and presents greater characteristics in adulthood. However, CALMs and other clinical features of the disease can occur during the early stages of development (Rasmussen *et al*., 2001; Tamura, 2021).

In this study, adults were the most commonly diagnosed, as symptoms appear and progress gradually over time (Rasmussen *et al*., 2001; Tamura, 2021). Consistent with our findings, one study showed that the median age at which diagnosis was sought was 19 years (Landry *et al*., 2021). Late diagnosis is also related to the low number of people seeking treatment in the early stages, due to a lack of information about the disease and the low education level of the population (Rasmussen *et al*., 2001; Wang *et al*., 2010). NF1 can usually be diagnosed by assessing the individual’s family history and by physical examination (Hirbe and Gutmann, 2014; Gutmann *et al*., 2017). Of the patients in the study, 44.3% had a history of NF1 in other family members. 

The presence of CALMs is the first and most common feature to be assessed (Ben-Shachar *et al*., 2017; Lalor *et al*., 2020). In our study, 92.7% of patients had CALMs, which is a predictive symptom for the diagnosis of NF1 (Lalor *et al*., 2020; Kehrer-Sawatzki and Cooper, 2022). Another common feature in patients with NF1 is axillary or inguinal freckling, which was present in more than 65% of individuals in our study. The results are in agreement with a study from 2020, which showed that axillary or inguinal freckling is present in 90% of individuals with NF1 (Miraglia *et al*., 2020). 

Diagnostic ocular manifestations may occur in early childhood, such as optic nerve glioma (OPG), or in late childhood and adolescence, such as LNs (Senthilkumar and Tripathy, 2023). OPGs tend to develop slowly, are more indolent in NF1 patients, and are generally asymptomatic (Bajenaru *et al*, 2005; Ferner *et al*, 2007). OPG had a low frequency (4.1%) in the present study, as only patient-reported complaints were included in the medical records, and most of these lesions are indolent. For LNs, 29.2% of patients had this characteristic. However, we observed a lower prevalence of LNs in the study population compared to data in the literature. LNs are considered the most typical clinical feature of the syndrome and are present in 95% of patients with NF1 (Iheanacho *et al*, 2022).

Neurofibromas are classified as benign tumours of the peripheral nerve sheath. They can cause local itching, pain, motor difficulties and sensory problems (Rad and Tee, 2016; Flores *et al*., 2022). In this study, 93.2% of patients had neurofibromas. A high frequency was also observed by Miraglia *et al* (2020), in which 78.1% of patients had some type of neurofibroma (Miraglia *et al*., 2020). In addition, individuals with NF1 have a high risk of developing neoplasms compared to the general population, with an incidence of 10% of cases (Eoli *et al*., 2019; Landry *et al*., 2021). In our study, 26.04% of NF1 patients developed neoplasms other than neurofibromas. Among the most common neoplasms in our study, MPNST, pheochromocytoma and central nervous system cancer were also the most frequent in a study carried out in 2016 (Landry *et al*., 2021). 

Alterations to bones were present in 62.5% of patients. These skeletal anomalies occur due to the absence of two copies of the NF1 gene in osteoclasts and osteoblasts, cells essential for bone resorption and formation, respectively (Hirbe and Gutmann, 2014). We found that 30.7% of the patients had scoliosis or more than one skeletal anomaly. According to the literature, the most common bone lesions are scoliosis, kyphosis and spinal deformity, with a range of prevalence of 8% to 60% (Larson *et al*., 2018). 

In a study carried out on rats, high *RAS* activity was correlated with increased GABA activity and changes in dopaminergic activity, which are associated with behavioural disorders such as attention deficit hyperactivity disorder, autism, dyslexia and cognitive difficulties (Gutmann *et al*., 2017; Bayat and Bayat, 2020; Kaczorowski *et al*., 2020). In the present study, 35 patients were found to have neurodevelopmental disorders, with males having the highest frequency. According to the literature, male patients are more prone to neurodevelopmental disorders (Jorge, 1995). Other neurological disorders, such as epilepsy and migraine, were also found in this study. Such disorders are frequent in individuals with NF1, such as migraine, which can be present in up to 33% of the cases (Pecoraro *et al*., 2017; Vogel *et al*., 2017; Miller *et al*., 2019).

## Conclusions

The study population with NF1 shows a high frequency of diagnostic features adopted by the NIH consensus, such as CALMs, axillary/inguinal ephelides, dermal neurofibromas and specific bone lesions, ophthalmological alterations, in addition to malignant and benign neoplasms and neurological disorders. The DataSUS database shows disparity in NF1 care in the North and Centre-West regions, as well as the low number of live births with the syndrome in the country. Therefore, due to the scarcity of data in public databases, the limitation of molecular tests and knowledge of health professionals in rare diseases, there is a need for new public policies for access to diagnoses, treatments and information about the disease for the Brazilian population.
